# Three-Dimensional Printing in Dentistry: Evolution, Technologies, and Clinical Application

**DOI:** 10.3390/polym18070785

**Published:** 2026-03-24

**Authors:** Citra Dewi Sahrir, Chin-Wei Wang, Yung-Kang Shen, Wei-Chun Lin

**Affiliations:** 1 School of Dentistry, College of Oral Medicine, Taipei Medical University, Taipei 110, Taiwan; d204112005@tmu.edu.tw (C.D.S.); jeffwa@tmu.edu.tw (C.-W.W.); 2 Department of Dental Material, Faculty of Dentistry, Hasanuddin University, Makassar 90245, Indonesia; 3 Division of Periodontics, Department of Oral Medicine, Taipei Medical University Hospital, Taipei 110, Taiwan; 4 School of Dental Technology, College of Oral Medicine, Taipei Medical University, Taipei 110, Taiwan; ykshen@tmu.edu.tw; 5 Department of Dentistry, Wan Fang Hospital, Taipei Medical University, Taipei 116, Taiwan

**Keywords:** additive manufacturing, 3D printing, digital dentistry, clinical application, vat photopolymerization

## Abstract

Three-dimensional (3D) printing, also known as additive manufacturing (AM), has become increasingly integrated into dentistry because of its high precision, efficiency, and ability to fabricate patient-specific devices. This review comprehensively discusses the historical development of 3D printing and outlines the fundamental principles of the most widely used technologies in dentistry, including stereolithography (SLA), digital light processing (DLP), and liquid crystal display (LCD). These technologies enable the accurate and efficient fabrication of dental models, crowns, bridges, dentures, surgical guides, orthodontic appliances, and tissue engineering scaffolds. Current clinical applications are systematically summarized across major dental disciplines, including prosthodontics, orthodontics, oral and maxillofacial surgery, endodontics, periodontics, and pediatric dentistry. Despite existing challenges, such as limited long-term clinical data for certain materials, high initial equipment costs, and post-processing requirements, 3D printing offers substantial advantages in terms of customization, workflow efficiency, and clinical predictability of the final product. Future developments in advanced biomaterials, artificial intelligence-assisted workflows, bioprinting, and four-dimensional (4D) printing are expected to further expand the role of additive manufacturing in personalized and regenerative dentistry.

## 1. Introduction

Three-dimensional (3D) printing, also referred to as additive manufacturing, has emerged as one of the most rapidly advancing technologies in modern manufacturing and healthcare industries [1,2]. The technology involves the fabrication of objects through the layer-by-layer deposition of materials, in which successive layers are formed upon previously solidified layers [3]. Unlike conventional subtractive manufacturing techniques, where material is removed from a solid block to achieve a desired shape, additive manufacturing constructs objects layer by layer directly from a digital 3D model, enabling the fabrication of complex geometries with high precision and minimal material waste [4]. In recent years, technological advancements in printing precision, digital design systems, and imaging technologies have expanded the use of 3D printing in healthcare [5]. Moreover, additive manufacturing offers broad application prospects in medical devices due to its ability to process a wide range of printable materials [6].

The conceptual foundation of 3D printing dates back to the 1980s, when Charles W. Hull introduced stereolithography and developed the first commercial 3D printer in 1986, marking a pivotal milestone in digital fabrication [6]. Since then, continuous technological advancements, particularly in computer-aided design and manufacturing (CAD/CAM), have accelerated the integration of 3D printing into dental applications. The ability to convert digital datasets derived from intraoral scanners, cone beam computed tomography (CBCT), or laboratory scans into physical objects has fundamentally transformed dental workflows [7].

The most widely used 3D printing technologies include stereolithography (SLA), digital light processing (DLP), liquid crystal display (LCD), and selective laser sintering (SLS), each of which offers distinct advantages depending on clinical requirements, material selection, and application [8,9]. These technologies support the fabrication of dental models, fixed and removable prostheses, surgical guides, orthodontic appliances, and tissue engineering scaffolds with high precision; however, previous studies have reported that their accuracy may be comparable to or, in certain applications, lower than that achieved by conventional subtractive manufacturing methods, depending on the material and fabrication parameters [10,11].

The integration of 3D printing into dental practice offers several clinical advantages, including reduced production time, enhanced customization, and improved fit and adaptation [12]. Moreover, 3D printing minimizes manual laboratory procedures, thereby reducing operator-dependent variability and enhancing the consistency of dental restorations and devices [13]. Consequently, 3D printing has been increasingly incorporated across multiple dental applications, including prosthodontics, oral and maxillofacial surgery, endodontics, periodontics, orthodontics, and pediatric dentistry.

Despite these advantages, challenges remain, such as limited long-term clinical data for certain printable materials, high initial equipment costs, and the need for post-processing steps that may affect the accuracy and strength [14,15]. Therefore, a comprehensive evaluation of the technological and material principles, clinical applications, and future potential of 3D printing in dentistry is essential.

Accordingly, this review primarily aims to (1) comprehensively outline the historical development of 3D printing technology relevant to dentistry, (2) outline the fundamental principles of commonly used additive manufacturing techniques and materials; and (3) critically summarize the current clinical applications of 3D printing across key dental disciplines, while highlighting existing limitations and future research directions, such as the integration of artificial intelligence (AI) and 4D printing, as well as the existing challenges and prospective trajectories that will shape the evolution of dental care in the future.

### Literature Search Methodology

To provide a comprehensive overview of the development and clinical applications of 3D printing in dentistry, a literature search was conducted using major scientific databases, including PubMed, Scopus, and Web of Science, supplemented by additional relevant sources identified through Google Scholar.

The search strategy included keywords such as “3D printing,” “additive manufacturing,” “digital dentistry,” “dental prostheses,” “bioprinting,” and “dental applications.” Publications addressing the technological principles, materials, and clinical applications of 3D printing in dentistry were considered.

Studies published primarily between 2000 and 2025 were reviewed to capture both the historical development and recent technological advancements in additive manufacturing for dental applications, with particular emphasis on studies published within the last decade. Earlier landmark studies were also included where relevant to provide historical context for the evolution of 3D printing technologies. Articles focusing on the technological principles, materials, and clinical applications of 3D printing in dentistry were included. Articles unrelated to dental applications or lacking sufficient relevance to the technological or clinical aspects of additive manufacturing were excluded.

## 2. Historical Development of 3D Printing in Dentistry

The history of 3D printing began with Dr. Hideo Kodama in 1981, who described a rapid prototyping system based on the principle of photopolymerization [16]. This concept was further developed by Charles W. Hull, who introduced SLA in 1986 and subsequently commercialized the first 3D printer, known as SLA-1, in 1988 [17,18]. These early innovations laid the foundation for modern additive manufacturing technology. In the 1990s, 3D printing expanded beyond industrial manufacturing to biomedical applications, including medicine and dentistry. The introduction of inkjet-based printing systems in 1993 enabled more precise material deposition and expanded the range of printable materials [19]. However, early dental applications were largely limited to anatomical models used for surgical planning, education, and communication, as the material properties and printing resolution were insufficient for intraoral use [20].

A major turning point occurred in the mid-2000s with the widespread adoption of CAD/CAM technology and the increasing availability of cone beam computed tomography (CBCT) [7]. The integration of digital imaging, virtual planning, and additive manufacturing has enabled the creation of patient-specific surgical guides and dental models with greater accuracy, significantly improving implant planning and maxillofacial surgical workflow [21,22].

From the 2010s, advances in printing accuracy, photopolymer resins, and metal-based printing technologies have facilitated the clinical expansion of 3D printing across various dental specialties [23]. Applications have expanded from diagnostic models to definitive restorations, orthodontic appliances, endodontic guides, custom implants, and maxillofacial reconstruction plates [24]. The publication of the ISO/ASTM 52900 standard in 2015 further supported the clinical translation of additive manufacturing by providing standardized terminology and process definitions, contributing to broader clinical acceptance and regulatory clarity [25].

Recently, the focus of 3D printing in dentistry has shifted toward personalized and regenerative approaches, including the development of bioactive scaffolds and patient-specific regenerative constructs [26]. Continuous advancements in materials science, digital workflows, and interdisciplinary collaboration have positioned 3D printing as a crucial component of contemporary digital dentistry, bridging the gap between virtual planning and clinical execution [12].

## 3. 3D Printing Process in Dentistry

**Figure 1:**
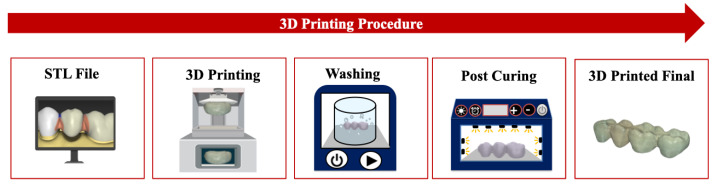
Overview of the 3D printing procedure in dentistry, illustrating STL file generation, additive manufacturing, washing, post-curing, and the resulting 3D-printed dental restoration.

The 3D printing process begins with the digital design of the intended object, which is typically created using CAD software or generated from scans of physical models [27]. This digital model was then converted and saved in the standard tessellation language (STL) format, which has been the most widely used file format for additive manufacturing since its introduction in 1987 [28]. Once the STL file was prepared, the fabrication process was initiated by transmitting digital data to a 3D printer. The printer deposits or solidifies raw material layer by layer to construct an object in three dimensions [29]. Each successive layer adheres precisely to the previous layer, enabling the formation of complex geometries with high accuracy [30]. This additive approach allows for rapid, material-efficient production with minimal waste [6]. An illustration of the 3D printing workflow is presented in Figure 1. This workflow also illustrates the integration of additive manufacturing with digital CAD/CAM systems commonly used in dentistry, where patient data acquired from intraoral scanners or CBCT imaging is converted into digital models for computer-aided design and subsequent 3D printing.

## 4. 3D Printing Technology and Material in Dentistry

Additive manufacturing technologies used in dentistry can generally be classified into several major categories, including vat photopolymerization, powder bed fusion, material extrusion, and material jetting systems [31]. Each printing technique operates using distinct fabrication principles and utilizes different classes of materials, which significantly influence the mechanical performance, accuracy, and clinical applicability of printed dental devices [32].

The selection and performance of materials used in additive manufacturing are critical considerations in dental applications [32]. Different 3D printing technologies utilize various material systems, including photopolymer resins, thermoplastics, ceramics, and metallic alloys, each presenting distinct mechanical properties and biological characteristics [23]. Mechanical performance, biocompatibility, and long-term stability are key factors influencing the clinical applicability of printed dental devices. Table 1 summarizes commonly used materials across different 3D printing technologies and highlights their mechanical characteristics, Biocompatibility, and typical dental applications.

In addition to material classification, the underlying curing mechanisms play a crucial role in determining the final properties of 3D-printed dental materials. In dental 3D printing, curing is predominantly based on photopolymerization [33], where light-activated photoinitiators trigger rapid crosslinking of resin monomers to form a solid polymer network [34]. Most vat photopolymerization systems, including SLA, DLP, and LCD, utilize photosensitive resins composed of methacrylate- or acrylate-based monomers, along with photoinitiators and functional additives that regulate viscosity, polymerization kinetics, and final material properties [35].

From a materials perspective, commonly used resin systems include dimethacrylate-based polymers such as bisphenol A-glycidyl methacrylate (Bis-GMA), urethane dimethacrylate (UDMA), and triethylene glycol dimethacrylate (TEGDMA), which exhibit favorable mechanical strength, biocompatibility, and printability for dental applications [36]. The final mechanical properties of printed dental materials, including flexural strength, hardness, and dimensional stability, are strongly influenced by monomer composition, filler content, degree of conversion, and post-curing conditions [14,15]. Therefore, a comprehensive understanding of both curing mechanisms and material composition is essential for optimizing the performance and clinical reliability of 3D-printed dental.

**Table 1:** Mechanical characteristics and material considerations of commonly used 3D printing technologies in dentistry.

Printing Technology	Materials Type	Mechanical Properties	Biocompatibility	Dental Application	References
Stereolithography (SLA)	Photopolymerized resin, plastics and ceramics	High flexural strength	Potential residual monomer release; requires proper post-curing	Dental models, surgical guides, provisional restorations, acrylic teeth, mouth guard, bite plane appliances	[37,38,39,40,41]
Digital Light Processing (DLP)	Photopolymerized resin, plastics and ceramics	Moderate to high flexural strength	Biocompatibility depends on resin composition and curing efficiency	Crowns, bridges, surgical guides, orthodontic models	[37,40,41,42,43]
Liquid Crystal Display (LCD)	Photopolymerized resin	Moderate Flexural Strength	Proper polymerization required to reduce cytotoxicity	Diagnostic models, provisional restorations	[40,41,44,45]
Selective Laser Sintering (SLS)	Plastics, ceramics and metals, powder such as alumide, polyamide, polyurethane	High mechanical strength and durability	Good biocompatibility for polymer-based materials	Surgical models, framework prototypes, scaffold	[42,43,46]
Selective Laser Melting (SLM)	Metals (titanium, titanium alloys, cobalt chrome, stainless steel)	Excellent mechanical strength	Good biocompatibility for polymer-based materials	Implants, maxillofacial plates, frameworks, metal crown	[37,43,47]
PolyJet Printing (PP)	Photopolymerized resin	Moderate to high flexural strength	Good biocompatibility	High detail models, scaffolds, surgical guides	[37,48]
Fused Deposition Modelling (FDM)	Thermoplastics (PLA, ABS, PEEK, nylon)	PEEK high strength and durability	Thermoplastics (PLA, ABS, PEEK, nylon)	Educational models, surgical guides, custom tray	[37,49,50]

**Figure 2:**
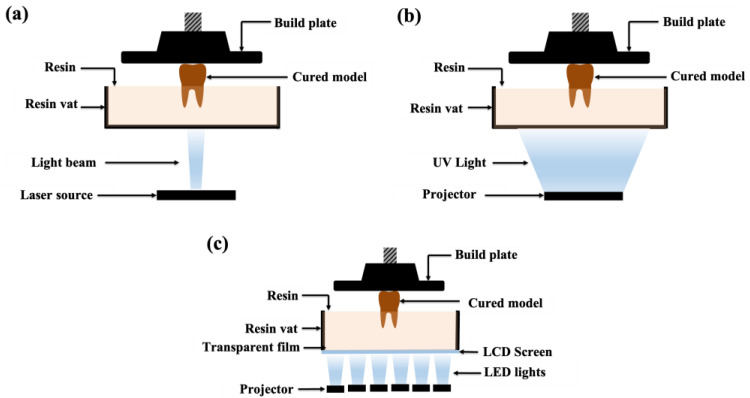
Schematic comparison of 3D printing technologies commonly used in dentistry: (**a**) SLA, (**b**) DLP, (**c**) LCD.

Among the various additive manufacturing techniques used in dentistry, vat photopolymerization technologies such as SLA, DLP, and LCD printing are among the most widely applied due to their high accuracy and suitability for dental materials, as illustrated in Figure 2. The following sections briefly describe the principles, advantages, and limitations of these commonly used technologies.

In addition to vat photopolymerization technologies, other additive manufacturing techniques such as SLS, SLM, PP and FDM are also utilized in biomedical and dental applications. SLS employs a laser to sinter powdered materials, including metal, polymers and ceramics, into solid structures and is commonly used for producing durable dental models and prosthetic components [6,46]. In contrast, SLM is designed for metal fabrication, using high-energy laser beams to fully melt metal powders, thereby enabling the production of dense and complex metallic frameworks and implants [46]. PolyJet printing differs significantly from powder-based systems, as the precise ejection of a liquid photopolymer onto the build platform is instantly cured by light, allowing for high-resolution fabrication with smooth and high precision [51]. Meanwhile, FDM represents a more accessible approach, relying on thermoplastic filament extrusion; however, its comparatively lower resolution and surface quality limit its application in precision-demanding dental workflows [51]. Nevertheless, the present review primarily focuses on vat photopolymerization techniques, as these methods are currently the most widely adopted in dentistry due to their superior accuracy, fine resolution, and compatibility with dental resin materials.

### 4.1. Stereolithography

The oldest and most popular 3D printing method in dentistry is the SLA. In this technique, a focused laser beam selectively polymerizes a liquid photopolymer resin within a resin vat in a layer-by-layer manner to form a solid structure [35]. Although SLA is generally slower than DLP, it remains a commonly used technology because of its high accuracy, fine resolution, and versatility in dental applications [8]. The advantages of SLA include excellent surface quality, high dimensional accuracy, and good mechanical properties of printed objects [52]. However, several limitations remain, such as relatively high equipment costs, mandatory post-processing steps, including washing and post-curing, and material restrictions, as SLA is primarily limited to polymer-based resins [53].

### 4.2. Direct Light Processing

DLP is based on the projection of ultraviolet (UV) light using a digital light projector to polymerize photopolymer resin [52]. Unlike SLA, which cures resin point by point using a laser, DLP projects the entire image of a layer simultaneously, significantly accelerating the printing process by curing all regions of a layer simultaneously [9].

DLP printers utilize a digital micromirror device (DMD) composed of numerous microscopic mirrors that selectively direct light onto the resin surface to define each layer [54]. This simultaneous curing mechanism allows DLP to achieve faster printing speeds while maintaining high accuracy, making it particularly suitable for dental models, crowns, and surgical guides [38]. Similar to SLA, printed objects produced using DLP require post-processing steps, such as washing and post-curing, to achieve optimal mechanical and dimensional properties [55].

### 4.3. Liquid Crystal Display

LCD 3D printing employs an LCD panel as a mask to selectively block or transmit light from an array of light-emitting diodes (LEDs) to cure a photopolymer resin [44]. The LCD panel enables parallel exposure of the resin surface without the need for optical lenses or complex projection systems, resulting in a simplified printer design [9,44]. One of the main advantages of LCD printing is its relatively low cost compared to DLP systems, as it uses more affordable components, making it an attractive low-cost alternative for dental applications [10]. However, LCD printing has some limitations. The rearrangement of liquid crystal molecules under an electric field may be incomplete during rapid switching, leading to mild light leakage and reduced curing precision [40]. Consequently, LCD printers generally exhibit lower accuracy and resolution than DLP systems, which may affect the dimensional fidelity of printed dental devices [9,11].

## 5. Clinical Application of 3D Printing in Dentistry

**Figure 3:**
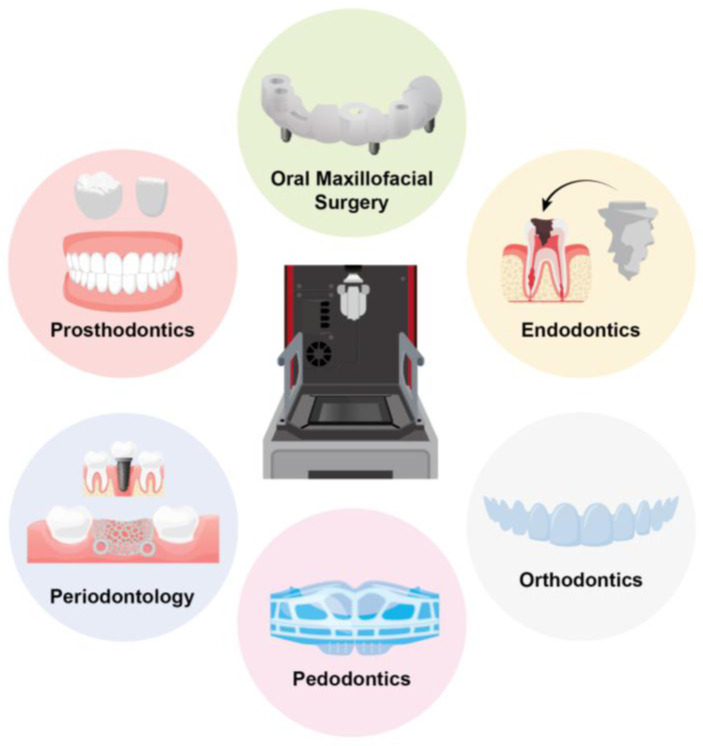
Overview of the clinical applications of 3D printing technology in dentistry. The arrow indicates the treatment process in endodontic procedures.

The wide range of clinical applications of 3D printing technology in dentistry is summarized in Figure 3, including its use in prosthodontics, oral and maxillofacial surgery, endodontics, periodontology, orthodontics, and pedodontics.

### 5.1. Prosthodontics

Prosthodontics is a critical dental discipline that focuses on restoring oral function and aesthetics by replacing missing teeth. Technological advancements, particularly in 3D printing, have revolutionized the field, allowing prosthodontists and dental technicians to create high-quality, customized prostheses with precise anatomical replication, replacing traditional methods [56,57]. Through the combination of CAD/CAM design and additive manufacturing, complex prostheses can be produced with enhanced accuracy, reduced fabrication time, and greater consistency [13]. Although challenges related to material durability, cost, and surface accuracy persist, ongoing advancements in materials science and printer technology are expected to expand the clinical utility of 3D printing in prosthodontics [56]. Given its rapid adoption, 3D printing has influenced several aspects of prosthodontic rehabilitation. The following subsections summarize its major applications:

- Crowns and bridges
  Traditionally fabricated dental restorations have the reliability and precision of restorations that are affected by human errors [8]. The adoption of 3D printing enables the production of crowns and bridges with high dimensional accuracy, superior marginal adaptation, and consistent esthetic results [57]. Tahayeri et al. reported that 3D-printed crowns and bridges demonstrated clinically acceptable mechanical properties [58]. Building on this material-based evidence, Kharat et al. confirmed that significant differences exist between 3D-printed and conventionally fabricated restorations, with 3D-printed crowns showing superior prosthesis fit, esthetics, occlusal stability, patient comfort, and overall patient satisfaction [59]. These findings highlight the growing clinical reliability of additively manufactured fixed prostheses in dentistry.

- Interim restorations
  Interim restorations play an essential role in fixed prosthodontics by protecting prepared teeth, maintaining occlusion and esthetics, and supporting soft tissue contours during treatment [60,61]. Conventional indirect fabrication methods require additional processes, while the accuracy decreases, and chair times increase due to the impression and model-making processes [62]. In contrast, 3D-printed interim restorations follow a fully digital workflow from intraoral scanning to computer-aided design and additive fabrication, which minimizes procedural steps and improves precision [8]. Hougne et al. reported a 98% survival rate of 3D-printed temporary crowns in a retrospective cohort study, with patients exhibiting significant improvements in esthetic satisfaction and oral health–related quality of life [63]. Similarly, Liu et al. confirmed that digitally printed interim crowns demonstrated marginal gaps 4.3 times smaller than those produced conventionally and reduced fabrication time from 10 min to 5 min [64]. These results support the clinical feasibility and efficiency of 3D-printed provisional restoration.

- Complete and partial removable dentures
  Conventional complete dentures have long been the standard treatment for edentulous patients [65]; however, they require multiple visits and extensive laboratory steps [66]. Despite their reliability, they may lack the precision and fit expected by patients, who increasingly demand greater comfort and function [67]. The introduction of CAD/CAM workflows and 3D printing has significantly improved denture fabrication by allowing precise digital design, better adaptation to mucosal surfaces, and enhanced mechanical properties of the printed denture base [68]. Casucci et al. demonstrated that 3D-printed removable dentures offer a practical and efficient alternative to traditional methods, reducing laboratory costs, chairside time, and the number of required appointments [65]. Furthermore, Goodacre et al. reported that patients fitted with digitally fabricated complete dentures experienced superior adaptation and reduced post-insertion adjustments compared with conventional dentures [69]. These advantages support the expanding role of additive manufacturing in the management of edentulous patients.

- Custom Trays
  Custom trays are individualized trays essential for taking impressions, registering temporary restorations, and registering bites. Conventionally, they are made from silicone from the patient’s teeth and require extremely high precision owing to the patient’s anatomy [70,71]. 3D printing offers a more accurate and faster method for producing these devices, and digital oral cavity scanning can also be used to create custom-designed trays [56]. Keshkiea et al. reported that 3D-printed custom trays demonstrated superior adaptability and reproducibility compared with manually fabricated trays because of their consistent thickness and precise design [72]. Deng et al. further confirmed that digitally fabricated diagnostic dentures reduce the number of patient visits, thereby increasing efficiency and improving clinical workflow. In addition, Sadr et al. showed that 3D-printed trays achieved better space distribution than conventional trays [73]. These findings illustrate the clinical value of additive manufacturing for impression tray fabrication.

### 5.2. Oral and Maxillofacial Surgery

In oral and maxillofacial surgery (OMFS), 3D printing technology has contributed to enhanced precision, predictability, and reduced surgical duration. It facilitates the training and practice of surgeons, improves communication with patients, and leads to more favorable surgical outcomes [74]. 3D printing in the treatment of OMFS patients was first reported by Brix et al., as cited in Dadhich et al. [17] and later popularized by Mankovich et al. in 1990, who proposed a new method of stereolithography for the production of anatomical models that promises to display full internal detail in 3D anatomical displays [20]. Today, additive manufacturing plays an essential role in the diagnosis, planning, and treatment of complex maxillofacial cases.

- 3D Printing in Management of Facial Trauma
  Facial trauma, especially when involving comminuted fractures, often forces surgeons to work through anatomical uncertainties [75]. Conventional intraoperative plate bending is time-consuming, less precise, and often requires multiple adjustments. 3D-printed pre-contoured implants improve accuracy and reduce the surgical workload [76]. Masada et al. demonstrated that 3D-printed fracture models improved operative planning, reduced plate contouring time, and resulted in better postoperative symmetry than the conventional method [77]. A similar finding was reported by Chakravarthy et al. in mandibular fractures. Using 3D-printed titanium plates tailored to the patient’s anatomy, surgeons no longer need to bend plates during surgery, saving operative time and reducing human error [78].

- 3D Printing in Orthognathic Surgery
  Traditional treatment planning is limited by projection and identification errors, especially in patients with facial asymmetry [79]. With the introduction of 3D printing, this planning paradigm has shifted. Surgeons now begin with a virtual surgical plan that simulates skeletal movements with a millimetric precision [80]. Lin et al. confirmed through a systematic review that the use of 3D printing methods in orthognathic surgery provides the benefit of optimal functional and aesthetic results, patient satisfaction, and precise translation of the treatment plan [80]. Clinical reports have shown that patients experience more symmetrical outcomes and faster functional recovery, while surgeons spend less time making intraoperative corrections [81].

- 3D Printing in Maxillofacial Tumor Resection and Reconstruction
  Reconstruction after maxillofacial tumor resection represents one of the greatest challenges in OMFS [82]. Old reconstruction methods, such as intraoperatively shaped titanium mesh and autologous bone grafting, frequently lack precision and long-term efficacy [83]. The integration of 3D printing in maxillofacial surgery facilitates the creation being customized to the unique anatomy of each patient, enhancing surgical accuracy, cosmetic results, and functional rehabilitation [84]. Li et al. reported a study of eight patients with different degrees of maxillofacial deformity who underwent orthognathic surgery using 3D-printed personalized titanium implants for the maxillary [85]. Abdelhamid et al. reinforced these findings in a randomized clinical trial involving Le Fort I osteotomies. Both 3D-printed splints and customized titanium plates transferred the virtual plan reliably, but the patient-specific plates performed even better, reducing operative time by eliminating the need for splints [86]. The result was a cleaner surgery, smoother workflow, and greater precision in anteroposterior positioning.

- 3D Printing in Total Joint Replacement
  Conventional temporomandibular joint (TMJ) prostheses often require intraoperative reshaping and often fail to adequately accommodate craniofacial asymmetries. In contrast, 3D printed patient-specific total joint replacement (TJR) prostheses enable precise anatomical adaptation [87], improved surgical accuracy, and more predictable functional outcomes, particularly in conjunction with computer-assisted surgery and virtual surgical planning [88]. Zheng et al. further demonstrated that 3D printed combined prostheses may serve as a viable alternative to established reconstruction techniques for temporomandibular joint and mandibular defects [87]. Parallel advancements have also been observed in conservative TMJ therapy, particularly in the application of repositioning splints fabricated using additive manufacturing. Jin et al. demonstrated that 3D printed repositioning splints are superior to conventional acrylic splints in reducing pain and improving mouth opening, primarily due to their ability to accurately replicate patient-specific occlusal and condylar anatomy [89]. Recent advances in additive manufacturing have also enhanced diagnostic accuracy and treatment planning in complex dental procedures, supporting more precise and individualized interventions [90]. Finally, Mercuri et al. reported that 3D printing technology has been increasingly adopted in the fabrication of metal TMJ components, progressively replacing conventional alloplastic systems and demonstrating significant potential for future clinical integration [91].

### 5.3. Endodontics

Endodontic treatment is often challenging because of anatomical variations, such as calcified or missing pulp canals, where hard tissue deposition complicates canal location and negotiation [90]. The introduction of AM technology has substantially improved diagnostic visualization, procedural planning, and treatment accuracy in complex cases [91]. CAD/CAM technology was first introduced by Duret and Preston in 1991 for restorative dentistry [92], and the adoption of 3D printing in endodontics accelerated following the publication of the ISO/ASTM 52900 standard in 2015 [93]. As previously discussed, precise visualization of internal tooth morphology is fundamental for predictable outcomes, and AM integrates radiographic information into patient-specific digital workflows that enhance clinical decision-making and execution [93]. The following subsections outline key applications of 3D printing in endodontics

- Guided Endodontic Access
  Access cavity preparation is one of the most critical phases of endodontic therapy because deviations during this step may lead to perforation or excessive dentin [94]. Guided endodontic access using 3D-printed templates enhances procedural accuracy by directing the bur toward the canal along a preplanned trajectory derived from CBCT and intraoral scans. Ackerman et al. demonstrated that digitally guided endodontic microsurgery significantly reduced length and angle deviations during apical resection compared with freehand procedures [95]. Similarly, Zhao et al. reported that 3D-printed surgical guides reliably improved the precision of apical access by stabilizing both the entry point and angulation during apicoectomy [96]. Collectively, these studies confirm that guided access minimizes iatrogenic complications commonly seen with conventional techniques.

- Autotransplantation
  In traditional autotransplantation, the donor tooth is repeatedly inserted into the prepared socket to assess fit, a process that increases extraoral time and risks damage to the periodontal ligament [97,98]. Systematic reviews report success rates of 80–91%, with 1 year survival rates of 97.4–98.0% and 5-year survival rates between 81–98.2% [99,100]. The integration of 3D-printed donor tooth replicas and surgical guides has markedly improved the procedure. In a case report, Sato et al. used a 3D-printed hemisection guide and tooth replica to transplant fused teeth in a young patient who was unsuitable for implants or fixed prosthetics. The digital workflow enhances socket preparation accuracy and reduces donor tooth manipulation, contributing to favorable clinical outcomes [101]. Compared with conventional methods, replica-assisted autotransplantation reduces operative uncertainty and supports biological preservation.

- Apicoectomy (Endodontic Microsurgery)
  Endodontic microsurgery (EMS) aims to achieve precise root-end access while minimizing bone removal and preventing injury to adjacent anatomical structures [102]. The incorporation of CAD/CAM-generated and 3D-printed surgical guides into EMS enables highly accurate osteotomy and root-end resection compared with traditional freehand surgery [103]. Giacomino et al. reported that 3D-printed templates provided accurate surgical pathways while reducing the risk of damaging vital structures during apicoectomy [104]. Hawkins et al. further demonstrated that guided EMS achieved optimal osteotomy geometry, root-end resection volume, and bevel angle, outperforming conventional freehand microsurgery [105]. These findings underscore the value of additive manufacturing in standardizing and improving the precision of microsurgery.

- Guided Post Removal
  Removing fiber or metal posts presents substantial clinical risks due to the possibility of perforation, excessive dentin loss, and structural compromise, complications frequently encountered with freehand rotary or ultrasonic techniques [106]. The use of 3D-printed static guides significantly improved the safety and predictability of post-removal. Multiple reports have described high success rates using guides that align the bur with the post while restricting lateral deviation [107]. Wu et al. demonstrated that 3D-printed resin guide plates enhanced procedural stability, reduced post-removal time, and increased safety compared to microscope-assisted ultrasonic techniques [108].

Overall, the integration of additive manufacturing and digital planning into endodontic procedures has demonstrated clinically meaningful improvements in procedural accuracy compared with conventional approaches. Guided endodontic access integrates CBCT imaging, intraoral scanning, and CAD/CAM planning to design patient-specific templates that direct the bur along a preplanned trajectory [109]. Using SLA, DLP, or PolyJet 3D printing technologies, these guides are fabricated from biocompatible resin materials that provide excellent dimensional accuracy, often within approximately 0.2 mm of the planned trajectory [110]. Previous studies have also reported that guided endodontics improves the accuracy of canal localization and reduces the risk of perforation and excessive dentin removal compared with conventional freehand techniques, particularly in cases of pulp canal obliteration [111].

### 5.4. Periodontics

Periodontal regeneration is challenging because of the multi-tissue nature of the periodontium, which consists of soft (gingiva and periodontal ligament) and hard (cementum and bone) tissues [112]. Over the past decade, this approach has gradually shaped the direction of periodontal research, especially as 3D printing has made it possible to create individualized scaffolds rather than relying solely on conventional graft materials [113]. Rasperini et al. reported the first clinical application of a patient-specific 3D-printed scaffold for periodontal regeneration, as cited in Sufaru et al. [114], establishing 3D-printed constructs as a promising strategy for various regenerative procedures.

- 3D Printing in Scaffolds in Periodontal Defects
  Advances in 3D printing now allow the fabrication of scaffolds with region-specific microarchitectures that replicate the biological needs of the periodontal ligament (PDL), cementum, and alveolar bone [115]. When combined with suitable growth-promoting agents, these engineered structures can support coordinated periodontal regeneration [116]. Furthermore, layer-by-layer 3D printing enables the production of patient-specific scaffolds precisely shaped to match the anatomy of individual periodontal defects, improving fit, stability, and regenerative outcomes [6]. In clinical applications, 3D printing scaffolds constitute an interesting alternative to traditional periodontal regeneration techniques [117]. Davidopoulou et al. similarly demonstrated that multidimensional 3D-printed scaffolds offer regenerative advantages in intrabony periodontal defects [117].

- 3D Printing in Socket Preservation
  Following tooth extraction, significant reductions in ridge width and height occur because of natural alveolar bone resorption [118]. The use of 3D-printed scaffolds has been introduced to maintain ridge dimensions [119]. Lee et al. demonstrated the successful augmentation of a severely atrophic mandibular ridge using a particulate bone graft placed within a patient-specific 3D-printed polycaprolactone/bioactive glass 7 (PCL/BGS7) scaffold [120]. Mangano et al. reported improved healing and controlled resorption when using a 3D-printed biphasic calcium phosphate (BCP) scaffold for bone augmentation [121]. Ghon et al. showed that using a 3D-printed PCL scaffold in extraction sockets supported normal bone healing compared to without using 3D printing [122].

- 3D Printing in Sinus and bone augmentation
  The primary challenge of implant placement in the posterior maxilla is the limited bone height resulting from maxillary sinus pneumatization [123]. CAD/CAM and 3D-printing technologies facilitate the fabrication of customized grafting materials for complex alveolar ridge augmentation procedures [124]. Mangano et al. evaluated a custom-made 3D-printed synthetic bone substitute for sinus augmentation and reported favorable outcomes [125]. Somji et al. further demonstrated that the anatomical variability of the maxillary sinus makes 3D-printed models highly valuable for pre-surgical planning [126]. In addition, Previous studies have reported that maxillary sinus pneumatization occurs following tooth extraction, particularly after maxillary second molars, and should be considered during implant planning in the posterior [123,127].

- 3D Printing for Guided Implant Placement Root-Analogue Implants
  The precision of 3D printing enables the fabrication of complex implant geometries, including root-analog implants that closely replicate natural tooth morphology [51]. These custom-designed Root-analogue Implants (RAIs) aim to improve socket fit and enhance primary stability, ultimately supporting better osseointegration [128]. Moin et al. demonstrated that current DLP-based 3D-printing systems can preemptively fabricate a one-piece zirconia root analog implant, highlighting the feasibility of this approach for guided implant placement [129].

### 5.5. Orthodontics

Rapid advances in 3D technology have enabled orthodontists to optimize their time and knowledge, delivering superior treatment outcomes through the digital design and 3D printing of orthodontic appliances [130]. Historically, Sassani and Roberts reported the feasibility of fabricating semi-automated orthodontic appliances using early computer-assisted technologies, although certain components still required manual assembly [131]. This early work established the foundation for fully digital orthodontic treatment. In 2008, Lauren and McIntyre introduced the first commercially produced computer-designed occlusal splints, marking the beginning of clinically integrated digital orthodontics [132]. Orthodontic treatment is often time-consuming and technique-sensitive, and is facilitated by 3D printing in a wide range of orthodontic applications [16]. The following subsections summarize the key clinical applications.

- Digital Orthodontic Models
  Conventional plaster models are susceptible to fractures and dimensional changes and require long-term physical storage. In contrast, digital models created via intraoral scanning and reproduced through 3D printing provide superior dimensional stability and easy archiving [133]. Brown et al. demonstrated that 3D-printed models exhibit greater accuracy than stone models in orthodontic analysis [134]. Similarly, Richard et al. and Tseng et al. showed that digital workflows enable rapid model production, eliminating the errors associated with impressions and plaster handling [45]. The ability to reproduce models on demand enhances efficiency, reduces storage requirements, and facilitates interdisciplinary consultation [45].

- Clear Aligners
  Clear aligners are comprehensive, specially designed removable orthodontic treatment appliances that are efficient for mild to moderate malocclusions [135]. In 1997, Align Technology introduced CAD/CAM-based aligner therapy into clinical practice, accelerating its global adoption [136]. Bae et al. reported good mechanical performance of 3D-printed aligner materials, supporting their use for precise tooth movements [137]. The integration of 3D printing enables the rapid production of sequential models, refinement stages, and customized aligner geometries, ultimately driving innovation in aligner fabrication and materials science [138].

- Retainers
  Retention after orthodontic treatment is a very important phase in the treatment that aims to keep teeth in their corrected positions [139]. CAD systems and 3D printing have also introduced new tools and materials for the 3D printing fabrication of retainers [140]. Beretta et al. demonstrated that retainers fabricated from polyetheretherketone (PEEK) via milling or molding exhibited clinically acceptable performance [141]. Cole et al. confirmed that 3D-printed retainers provide a fit accuracy comparable to that of thermoformed retainers [142]. Additionally, Win et al. reported the favorable mechanical stability of a 3D-printed hemielliptcal retainer design, supporting its feasibility for intraoral use [143].

- Customized Orthodontic Brackets
  Orthodontists use commercial straight wire brackets, whereas custom brackets are preferred for lingual orthodontic treatment [144]. AM allows the production of patient-specific brackets designed to optimize tooth movement efficiency [145]. Nguyen et al. reported the successful management of a Class II malocclusion with arch length discrepancy using 3D-printed lingual appliances [146]. Alam et al. demonstrated that customized 3D-printed brackets improved treatment quality and reduced overall treatment duration compared with conventional brackets [147]. Hanson et al. further confirmed that 3D-printed brackets exhibit adequate shear bond strength for clinical application [148]. In addition, flexible or elastic 3D-printable resins have gained increasing attention for the fabrication of indirect bonding trays (IBTs) in orthodontics, as they allow improved adaptation to tooth morphology and facilitate accurate bracket positioning. Previous studies have demonstrated that 3D-printed IBTs can achieve high transfer accuracy within clinically acceptable limits, although variations in torque, tip, and operator experience may influence the final outcomes [149,150].

- Nasoalveolar Molding
  Nasoalveolar molding (NAM) therapy is essential for infants with cleft lip and palate, requiring carefully shaped appliances that guide alveolar segments before primary surgical repair [151]. 3D technological advancements have been employed to design NAM devices more efficiently and create objective, standardized means of measuring progressive morphological changes during therapy [152]. Puneet et al. reported that 3D-printed NAM aligners achieved over 90% accuracy in reducing alveolar cleft gaps [153]. Yu et al. further confirmed that CAD-based NAM therapy effectively narrows the alveolar cleft and improves maxillary sagittal length in affected infants [154].

### 5.6. Pedodontics

Pedodontics is a dental subspecialty focused on the care of children, requiring specialized skills in behavioral management due to unique challenges in treating young patients [155]. Advancements in 3D printing technology are providing a new direction in pediatric dentistry by offering innovative solutions to traditional challenges [156]. The following subsections outline the most relevant clinical applications in this field.

- Training and Simulation
  Conventionally, extracted human teeth are used for dental students training in tooth morphology and identification. However, in recent years, it has become possible to create interactive, digitally printable virtual models using 3D printing [157]. A study by Aktas et al. showed that 3D printed models contribute to a more comprehensive and structured educational process, creating not only theoretical knowledge but also hands-on experience [158]. Mello et al., in a systematic review study, also showed that 3D printing allows the creation of patient-specific models that replicate native anatomical structures more accurately, improve the quality of training, and can contribute to higher clinical success and reduce stress during patient care [159].

- Surgical Applications
  Pediatric surgery presents unique challenges, requiring a specialized approach due to the complexity of compact anatomy and the presence of distinct congenital features in young patients [160]. Les et al. reported successful implantation and demonstrated the initial clinical efficacy of a 3D-printed bioresorbable airway splint device in a cohort of critically ill children [161].

- Pediatric Oral Rehabilitation
  Children with structural abnormalities, developmental defects, or trauma-related tooth loss often require customized oral appliances to restore function and esthetics [24]. Additive manufacturing enables the fabrication of lightweight, child-specific prosthetic devices with excellent fit and comfort [162]. Song et al. demonstrated that 3D-printed resin is clinically feasible for pediatric provisional restorations, with material selection depending on occlusal load, patient age, and functional needs [163]. Agrawal et al., in their case report, presented the successful treatment of a 4-year-old patient with complete anodontia using 3D-printed complete dentures [164].

- Customized Space Maintainers
  Space maintainers are essential in pedodontics to maintain arch length after the premature loss of primary teeth [165]. Watson et al. showed that the retention of 3D printed space maintainers was significantly lower compared to traditional [166]. Thakur et al. also confirmed that 3D printed space retainers such as band and loop space retainers showed an impressive retention rate of 77.4% over 9 months compared to conventional at 51.6% [167].

## 6. Future Directions

### 6.1. Bioprinting of Dental Tissues

Bioprinting represents a promising future direction in dentistry by enabling the fabrication of scaffolds that incorporate living cells, bioactive molecules, and growth factors for tissue regeneration [168]. Advances in bioink formulation and printing precision have opened new possibilities for regenerating complex dental and periodontal tissues, including alveolar bone, periodontal ligament, and gingival structures [115]. Although current applications remain largely experimental, continued progress in biomaterials, vascularization strategies, and cell–material interactions may facilitate the clinical translation of bioprinted constructs for regenerative dental therapies [169].

### 6.2. Artificial Intelligence Assisted 3D Printing

AI is increasingly being integrated into digital dental workflows to enhance the efficiency, accuracy, and personalization of 3D printing processes [64]. AI-driven design optimization enables automated generation of patient-specific dental devices by analyzing anatomical data from intraoral scans and CBCT images [170]. In addition, AI-assisted systems support real-time error detection, tooth movement, custom aligner fabrication, optimization of treatment time, quality control, and predictive maintenance during printing [64,170]. These developments are expected to improve clinical reliability and streamline chairside and laboratory workflows in dentistry. Recent studies have demonstrated that AI-designed dental restorations can achieve comparable trueness and marginal fit to human-designed restorations, while significantly reducing design time (up to 4–9 times faster), thereby enhancing both efficiency and clinical applicability [64].

### 6.3. Four-Dimensional Printing

Four-Dimensional (4D) printing extends conventional 3D printing by incorporating smart materials capable of responding to external stimuli such as warm water, light, and heat to execute diverse functionalities or mechanical forces [171]. In dentistry, this technology has the potential to enable adaptive dental devices, including orthodontic appliances and prosthetic components that can change shape or function over time in response to physiological conditions [172,173]. Although still in an early stage of development, 4D printing may offer innovative solutions for dynamic and patient-responsive dental treatments in the future.

### 6.4. Integration of 3D Printing with Virtual and Augmented Reality

The integration of 3D printing with virtual reality (VR) and augmented reality (AR) technologies represents an emerging trend in digital dentistry [174]. VR and AR can enhance treatment planning, surgical simulation, and dental model education by enabling interactive visualization of patient-specific anatomy and planned procedures [175]. When combined with 3D printing, these technologies may improve preoperative planning accuracy, clinician training, and patient communication, ultimately supporting more predictable and efficient clinical outcomes [175,176].

## 7. Limitations

Despite the increasing adoption of 3D printing technologies in dentistry, several limitations remain. Although numerous studies report improved efficiency and customization in dental applications, long-term clinical evidence regarding the durability and biological safety of many printable dental materials remains limited [57,177]. Concerns related to residual monomer release, polymer degradation, and long-term biocompatibility of photopolymer-based resins require further investigation through longitudinal clinical studies. In addition, the present review primarily focuses on the technological development and clinical applications of 3D printing in dentistry. A more detailed discussion of the material science aspects, including polymer composition, polymerization mechanisms, and mechanical properties of printable dental polymers, was beyond the primary scope of this review. Future studies should further investigate these material-related aspects to better understand their influence on mechanical performance, biocompatibility, and long-term clinical outcomes.

Furthermore, the current literature demonstrates considerable methodological variability, including differences in printer types, material formulations, printing parameters, and post-processing protocols. This heterogeneity makes direct comparisons between studies challenging and highlights the need for standardized experimental protocols and more robust clinical investigations.

Economic factors should also be considered when evaluating the broader clinical adoption of 3D printing technologies in dentistry. Compared with conventional fabrication techniques such as manual laboratory procedures or subtractive milling, additive manufacturing often requires relatively high initial investment costs for printers, software, and specialized training [178]. Nevertheless, digital workflows associated with 3D printing may reduce material waste, labor requirements, and production time, particularly for customized dental devices [6]. Consequently, although the initial setup cost may be relatively high, the overall economic feasibility of additive manufacturing may improve over time, depending on production volume and clinical application [6,179]. Further research is still needed to provide more comprehensive economic evaluations comparing additive manufacturing with conventional fabrication approaches in dental practice.

## 8. Conclusions

3D printing has become a transformative technology in dentistry. This review highlights the evolution of additive manufacturing and summarizes the clinical relevance of key 3D printing technologies. Across major dental disciplines, including prosthodontics, oral and maxillofacial surgery, endodontics, periodontics, orthodontics, and pedodontics, 3D printing has demonstrated significant advantages in precision, efficiency, customization, and workflow consistency. By reducing manual laboratory procedures, additive manufacturing supports more predictable clinical outcomes. Nevertheless, challenges remain related to material limitations, post-processing requirements, and regulatory standardization. Looking ahead, continued advances in biomaterials, bioprinting, artificial intelligence–assisted workflows, emerging 4D printing technologies, and the integration of virtual and augmented reality are expected to further expand the clinical potential of 3D printing. Interdisciplinary collaboration will be essential to ensure its safe, effective, and sustainable integration into future dental practice.

## Data Availability

No new data were created or analyzed in this study.

## Abbreviations

The following abbreviations are used in this manuscript:

3D	Three-dimensional
4D	Four-dimensional
AI	Artificial intelligence
AM	Additive manufacturing
AR	Augmented reality
BCP	Biphasic calcium phosphate
BGS7	Bioactive glass 7
Bis-GMA	Bisphenol A-glycidyl methacrylate
CAD	Computer-aided design
CAM	Computer-aided manufacturing
CBCT	Cone beam computed tomography
DLP	Digital light processing
DMD	Digital micromirror device
DoC	Degree of conversion
FDM	Fused deposition modeling
LCD	Liquid crystal display
LEDs	Light-emitting diodes
OMFS	Oral and maxillofacial surgery
PCL	Polycaprolactone
PEEK	Polyetheretherketone
PP	PolyJet printing
SLA	Stereolithography
SLM	Selective laser melting
SLS	Selective laser sintering
STL	Standard tessellation language
TEGDMA	Triethylene glycol dimethacrylate
TMJ	Temporomandibular joint
UDMA	Urethane dimethacrylate
UV	Ultraviolet
VR	Virtual reality

## References

[1] Buchanan C., Gardner L. (2019). Metal 3D printing in construction: A review of methods, research, applications, opportunities and challenges. Eng. Struct..

[2] Gadagi B., Lekurwale R. (2021). A review on advances in 3D metal printing. Mater. Today Proc..

[3] Loges K., Tiberius V. (2022). Implementation Challenges of 3D Printing in Prosthodontics: A Ranking-Type Delphi. Materials.

[4] Nesic D., Schaefer B.M., Sun Y., Saulacic N., Sailer I. (2020). 3D Printing Approach in Dentistry: The Future for Personalized Oral Soft Tissue Regeneration. J. Clin. Med..

[5] Bhatt S., Simre S., Patadiya H., Yadav D., Deepti B., Sathvik S., Manamasa Y. (2025). Review Article 3D Printing in Dentistry: A Review. J. Adv. Med. Dent. Sci. Res..

[6] Chen Y., Wei J. (2025). Application of 3D Printing Technology in Dentistry: A Review. Polymers.

[7] Chander N.G., Gopi A. (2024). Trends and future perspectives of 3D printing in prosthodontics. Med. J. Armed Forces India.

[8] Schweiger J., Edelhoff D., Güth J.-F. (2021). 3D printing in digital prosthetic dentistry: An overview of recent developments in additive manufacturing. J. Clin. Med..

[9] Tsolakis I.A., Papaioannou W., Papadopoulou E., Dalampira M., Tsolakis A.I. (2022). Comparison in Terms of Accuracy between DLP and LCD Printing Technology for Dental Model Printing. Dent. J..

[10] Lo Giudice A., Ronsivalle V., Rustico L., Aboulazm K., Isola G., Palazzo G. (2022). Evaluation of the accuracy of orthodontic models prototyped with entry-level LCD-based 3D printers: A study using surface-based superimposition and deviation analysis. Clin. Oral Investig..

[11] Quan H., Zhang T., Xu H., Luo S., Nie J., Zhu X. (2020). Photo-curing 3D printing technique and its challenges. Bioact. Mater..

[12] Borthakur P.P., Sahariah J.J., Sarma M., Das A., Pathak K., Ahmad M.Z., Abdel-Wahab B.A. (2025). Advances in 3D printing for dentistry: Clinical applications and future perspectives. Explor. Med..

[13] Figueiredo-Pina C.G., Serro A.P. (2023). 3D Printing for Dental Applications. Materials.

[14] Sahrir C.D., Lin W.-S., Wang C.-S., Lin H.-E., Wang C.-W., Lin W.-C. (2025). Effects of 3D-printers and manufacturer-specified post-curing units on the dimensional accuracy, compressive strength, and degree of conversion of resin for fixed dental prostheses. J. Dent. Sci..

[15] Sahrir C.D., Lin W.S., Wang C.W., Lin W.C. (2024). Effects of post-curing light intensity on the trueness, compressive strength, and resin polymerization characteristics of 3D-printed 3-unit fixed dental prostheses. J. Prosthodont..

[16] Jambhule S., Palandurkar M., Shewale A. (2022). 3D Printing in Dentistry. Int. J. Adv. Res..

[17] Dadhich A., Nilesh K., Shah S., Saluja H. (2022). Three-dimensional printing in maxillofacial surgery: A quantum leap in future. Natl. J. Maxillofac. Surg..

[18] Chua C.K., Leong K.F., Lim C.S. (2010). Rapid Prototyping: Principles and Applications.

[19] Sonika S., Esther Nalini H., Renuka Devi R. (2023). Quintessential commence of three-dimensional printing in periodontal regeneration-A review. Saudi Dent. J..

[20] Mankovich N.J., Cheeseman A.M., Stoker N.G. (1990). The display of three-dimensional anatomy with stereolithographic models. J. Digit. Imaging.

[21] Zoabi A., Redenski I., Oren D., Kasem A., Zigron A., Daoud S., Moskovich L., Kablan F., Srouji S. (2022). 3D printing and virtual surgical planning in oral and maxillofacial surgery. J. Clin. Med..

[22] Singh T., Bhola N., Reche A. (2023). The Utility of 3D Printing for Surgical Planning and Patient-Specific Implant Design in Maxillofacial Surgery: A Narrative Review. Cureus.

[23] Yüceer Ö.M., Kaynak Öztürk E., Çiçek E.S., Aktaş N., Bankoğlu Güngör M. (2025). Three-dimensional-printed photopolymer resin materials: A narrative review on their production techniques and applications in dentistry. Polymers.

[24] Almarshadi R., Hamdi S., Hadi F., Alshehri A., Alsahafi R., Aljohani N., Alyamani R., Alhbchi M., Alqahtani N., Almutiri R. (2025). Redefining Digital Dentistry: Multidisciplinary Applications of 3D Printing for Personalized Dental Care. Cureus.

[25] (2015). Additive Manufacturing—General Principles—Terminology.

[26] Mobarak M.H., Islam M.A., Hossain N., Al Mahmud M.Z., Rayhan M.T., Nishi N.J., Chowdhury M.A. (2023). Recent advances of additive manufacturing in implant fabrication—A review. Appl. Surf. Sci. Adv..

[27] Pagac M., Hajnys J., Ma Q.-P., Jancar L., Jansa J., Stefek P., Mesicek J. (2021). A Review of Vat Photopolymerization Technology: Materials, Applications, Challenges, and Future Trends of 3D Printing. Polymers.

[28] Manoj Prabhakar M., Saravanan A.K., Haiter Lenin A., Jerin leno I., Mayandi K., Sethu Ramalingam P. (2021). A short review on 3D printing methods, process parameters and materials. Mater. Today Proc..

[29] Jandyal A., Chaturvedi I., Wazir I., Raina A., Ul Haq M.I. (2022). 3D printing—A review of processes, materials and applications in industry 4.0. Sustain. Oper. Comput..

[30] Trojan J., Trebuňa P., Svetlík J., Kopec J. (2025). Case Study: Component Design for Streamlining the Manufacturing Process Using 3D Printing. Processes.

[31] Huang S., Wei H., Li D. (2023). Additive manufacturing technologies in the oral implant clinic: A review of current applications and progress. Front. Bioeng. Biotechnol..

[32] Cai H., Xu X., Lu X., Zhao M., Jia Q., Jiang H.-B., Kwon J.-S. (2023). Dental materials applied to 3D and 4D printing technologies: A review. Polymers.

[33] Ebrahimi M., Shaikh H., Rezvani Sichani H., Ramachandran R.A., Paramasivan M., Fazle Alam M., Mezzomo L., Dubey N., Mathew M.T. (2026). Additive manufacturing for Dentistry: A comprehensive review of techniques and applications. Prog. Mater. Sci..

[34] Sahrir C.D., Ruslin M., Lee S.-Y., Lin W.-C. (2024). Effect of various post-curing light intensities, times, and energy levels on the color of 3D-printed resin crowns. J. Dent. Sci..

[35] Graça A., Bom S., Martins A.M., Ribeiro H.M., Marto J. (2024). Vat-based photopolymerization 3D printing: From materials to topical and transdermal applications. Asian J. Pharm. Sci..

[36] Valencia-Blanco O.J., Pérez-Pevida E., Robles-Cantero D., Montalvillo E., Gil J., Brizuela-Velasco A. (2025). Comparative Analysis of the Physicochemical Properties of 3D-Printed and Conventional Resins for Temporary Dental Restorations. Prosthesis.

[37] Brian N.A. (2021). 3D Printing Part 2—A Literature Review of 3D Printing Materials in Dentistry. Preprint.

[38] Beyer M., Scheller L., Burde A.V., Abazi S., Sommacal A., Seifert L., Sharma N., Thieringer F.M. (2025). Comparative Evaluation of SLA and DLP 3D Printing in Dental Implant Guides: Impact on Fabrication Accuracy, Speed, and Resin Usage. Dent. J..

[39] Balhaddad A.A., Garcia I.M., Mokeem L., Alsahafi R., Majeed-Saidan A., Albagami H.H., Khan A.S., Ahmad S., Collares F.M., Della Bona A. (2023). Three-dimensional (3D) printing in dental practice: Applications, areas of interest, and level of evidence. Clin. Oral Investig..

[40] Katheng A., Prawatvatchara W., Chaiamornsup P., Sornsuwan T., Lekatana H., Palasuk J. (2025). Comparison of mechanical properties of different 3D printing technologies. Sci. Rep..

[41] Chen H., Cheng D.-H., Huang S.-C., Lin Y.-M. (2021). Comparison of flexural properties and cytotoxicity of interim materials printed from mono-LCD and DLP 3D printers. J. Prosthet. Dent..

[42] Arcila L.V.C., de Carvalho Ramos N., Bottino M.A., Tribst J.P.M. (2020). Indications, materials and properties of 3D printing in dentistry: A literature overview. Res. Soc. Dev..

[43] Hilma N.S., Anad K.S., Sreekumar A.V. (2020). 3D printing in prosthodontics. Int. J. Recent Sci. Res..

[44] Tsolakis I.A., Gizani S., Panayi N., Antonopoulos G., Tsolakis A.I. (2022). Three-dimensional printing technology in orthodontics for dental models: A systematic review. Children.

[45] Tseng C.W., Lin W.S., Sahrir C.D., Lin W.C. (2025). The impact of base design and restoration type on the resin consumption, trueness, and dimensional stability of dental casts additively manufactured from liquid crystal display 3D printers. J. Prosthodont..

[46] Song Y., Ghafari Y., Asefnejad A., Toghraie D. (2024). An overview of selective laser sintering 3D printing technology for biomedical and sports device applications: Processes, materials, and applications. Opt. Laser Technol..

[47] Baciu E.-R., Cimpoeșu R., Vițalariu A., Baciu C., Cimpoeșu N., Sodor A., Zegan G., Murariu A. (2021). Surface Analysis of 3D (SLM) Co–Cr–W Dental Metallic Materials. Appl. Sci..

[48] Hems E., Knott N.J. (2014). 3D printing in prosthodontics. Fac. Dent. J..

[49] Chen H., Yang X., Chen L., Wang Y., Sun Y. (2016). Application of FDM three-dimensional printing technology in the digital manufacture of custom edentulous mandible trays. Sci. Rep..

[50] Moby V., Dupagne L., Fouquet V., Attal J.P., François P., Dursun E. (2022). Mechanical Properties of Fused Deposition Modeling of Polyetheretherketone (PEEK) and Interest for Dental Restorations: A Systematic Review. Materials.

[51] Alqutaibi A.Y., Alghauli M.A., Aljohani M.H.A., Zafar M.S. (2024). Advanced additive manufacturing in implant dentistry: 3D printing technologies, printable materials, current applications and future requirements. Bioprinting.

[52] Hassanpour M., Narongdej P., Alterman N., Moghtadernejad S., Barjasteh E. (2024). Effects of Post-Processing Parameters on 3D-Printed Dental Appliances: A Review. Polymers.

[53] Jawahar A., Maragathavalli G. (2019). Applications of 3D printing in dentistry–a review. J. Pharm. Sci. Res..

[54] Kadry H., Wadnap S., Xu C., Ahsan F. (2019). Digital light processing (DLP) 3D-printing technology and photoreactive polymers in fabrication of modified-release tablets. Eur. J. Pharm. Sci..

[55] Jafarpour D., El-Amier N., Tahboub K., Zimmermann E., Pero A.C., de Souza R. (2025). Effects of DLP printing orientation and postprocessing regimes on the properties of 3D printed denture bases. J. Prosthet. Dent..

[56] Alyami M.H. (2024). The Applications of 3D-Printing Technology in Prosthodontics: A Review of the Current Literature. Cureus.

[57] Rezaie F., Farshbaf M., Dahri M., Masjedi M., Maleki R., Amini F., Wirth J., Moharamzadeh K., Weber F.E., Tayebi L. (2023). 3D Printing of Dental Prostheses: Current and Emerging Applications. J. Compos. Sci..

[58] Tahayeri A., Morgan M., Fugolin A.P., Bompolaki D., Athirasala A., Pfeifer C.S., Ferracane J.L., Bertassoni L.E. (2018). 3D printed versus conventionally cured provisional crown and bridge dental materials. Dent. Mater..

[59] Kharat S., Dudhani S.I., Kouser A., Subramanian P., Bhattacharjee D., Jhamb V. (2024). Exploring the Impact of 3D Printing Technology on Patient-Specific Prosthodontic Rehabilitation: A Comparative Study. J. Pharm. Bioallied Sci..

[60] Kim E.K., Park E.Y., Kang S. (2023). Three-dimensional printing of temporary crowns with polylactic acid polymer using the fused deposition modeling technique: A case series. J. Yeungnam Med. Sci..

[61] Alkhateeb R.I., Algaoud H.S., Aldamanhori R.B., Alshubaili R.R., Alalawi H., Gad M.M. (2023). Fracture Load of 3D-Printed Interim Three-Unit Fixed Dental Prostheses: Impact of Printing Orientation and Post-Curing Time. Polymers.

[62] Alghauli M., Alqutaibi A.Y., Wille S., Kern M. (2024). 3D-printed versus conventionally milled zirconia for dental clinical applications: Trueness, precision, accuracy, biological and esthetic aspects. J. Dent..

[63] del Hougne M., Di Lorenzo I., Höhne C., Schmitter M. (2024). A retrospective cohort study on 3D printed temporary crowns. Sci. Rep..

[64] Liu C.M., Lin W.C., Lee S.Y. (2024). Evaluation of the efficiency, trueness, and clinical application of novel artificial intelligence design for dental crown prostheses. Dent. Mater..

[65] Casucci A., Ferrari Cagidiaco E., Verniani G., Ferrari M., Borracchini A. (2025). Digital vs. conventional removable complete dentures: A retrospective study on clinical effectiveness and cost-efficiency in edentulous patients: Clinical effectiveness and cost-efficiency analysis of digital dentures. J. Dent..

[66] Carlsson G.E. (2004). Responses of jawbone to pressure. Gerodontology.

[67] Fenlon M.R., Sherriff M. (2004). Investigation of new complete denture quality and patients’ satisfaction with and use of dentures after two years. J. Dent..

[68] Srinivasan M., Kalberer N., Naharro M., Marchand L., Lee H., Müller F. (2020). CAD-CAM milled dentures: The Geneva protocols for digital dentures. J. Prosthet. Dent..

[69] Goodacre B.J., Goodacre C.J., Baba N.Z., Kattadiyil M.T. (2016). Comparison of denture base adaptation between CAD-CAM and conventional fabrication techniques. J. Prosthet. Dent..

[70] Kunusoth R., Colvenkar S., Thotapalli S., Alwala A.M., Prakash R. (2022). Custom Sectional Impression Tray With Sectional Handle for Microstomia Patients. Cureus.

[71] Deng K., Chen H., Wang Y., Zhou Y., Sun Y. (2022). Evaluation of a novel 3D-printed custom tray for the impressions of edentulous jaws. J. Dent..

[72] Keshkiea N.M., Jnaid F.A. (2022). Evaluation of Custom Tray Fabricated by CAD/3D Printer Compared with Traditional Technique for Complete Denture. Teikyo Med. J..

[73] Sadr K., Ghasemi S., Garjan A. (2020). Adaptation of Custom Trays Fabricated Using CAD/3D Printer and Manual Techniques Adaptation of Custom Trays Fabricated Using CAD/3D Printer and Manual Techniques. Int. J. Pharm. Phytopharm. Res..

[74] Hadad H., Lima F., Shirinbak I., Porto T., Chen J., Guastaldi F. (2023). The Impact of 3D Printing on Oral and Maxillofacial Surgery. J. 3D Print. Med..

[75] Chew K.-Y., Kok Y.O., Pek W.S., Too C.W., Tan B.-K. (2021). Surgical planning using facial fracture 3D models: The role of cyanoacrylate glue and miniplating for anatomical reduction. JPRAS Open.

[76] Bertin E., Coussens C., Brumpt E., Meyer C., Louvrier A. (2025). 3D printing and acute maxillofacial trauma: An overview of the literature. 3D Print. Med..

[77] Masada K., Cristino D., Dear K., Hast M., Mehta S. (2023). 3-D Printed Fracture Models Improve Resident Performance and Clinical Outcomes in Operative Fracture Management. J. Surg. Educ..

[78] Chakravarthy C., Gawade K., Mathpati S., G N., V M. (2024). 3D Printed Maxillofacial Trauma Plates as a Valuable Alternative to Conventional Miniplates: A Case Report. Int. J. Innov. Res. Med. Sci..

[79] Lonic D., Pai B.C.-J., Yamaguchi K., Chortrakarnkij P., Lin H.-H., Lo L.-J. (2016). Computer-Assisted Orthognathic Surgery for Patients with Cleft Lip/Palate: From Traditional Planning to Three-Dimensional Surgical Simulation. PLoS ONE.

[80] Lin H.-H., Lonic D., Lo L.-J. (2018). 3D printing in orthognathic surgery—A literature review. J. Formos. Med. Assoc..

[81] Dong Z., Yang S., Zhao Y., Duan Y., Zheng C., Guo L., Lyu J. (2025). Improved perioperative outcomes and early functional recovery with 3D-printed osteotomy guide plates in ulnar shortening osteotomy: A retrospective study. J. Exp. Orthop..

[82] Memon A.R., Wang E., Hu J., Egger J., Chen X. (2020). A review on computer-aided design and manufacturing of patient-specific maxillofacial implants. Expert Rev. Med. Devices.

[83] Du R., Su Y.-X., Yan Y., Choi W.S., Yang W.-F., Zhang C., Chen X., Curtin J.P., Ouyang J., Zhang B. (2020). A Systematic Approach for Making 3D-Printed Patient-Specific Implants for Craniomaxillofacial Reconstruction. Engineering.

[84] Yousif E., Abdalla M., Jumaa H., Fadlelmola S., Haboura O., Abdelrahim A., Elsayed A.A., Abdelrahim M.A. (2025). 3D-Printed Patient-Specific Implants in Maxillofacial Reconstruction: A Systematic Review and Meta-Analysis of Clinical Outcomes and Workflow Efficiency. Cureus.

[85] Li B., Jiang T.F., Cai M., Shen S., Jiang W., Shen G., Wang X. (2016). Accuracy of 3D printing individual titanium plates for maxillary repositioning in orthognathic surgery. China J. Oral Maxillofac. Surg..

[86] Abdelhamid A.M., Hassan A.M., El-Mohandes W.A. (2025). Accuracy Assessment of Customized Titanium Plates Compared to 3D-Printed Splints in Le Fort I Osteotomy: A Randomized Clinical Trial Evaluating Clinical and Radiographic Outcomes. Cureus.

[87] Zheng J., Huo L., Jiao Z., Wei X., Bu L., Jiang W., Luo Y., Chen M., Yang C. (2024). 3D-printed temporomandibular joint-mandible combined prosthesis: A prospective study. Oral Dis..

[88] Gomez N., Boccalatte L., Ruiz Á., Nassif M., Figari M., Ritacco L. (2020). Total Temporomandibular Joint Replacement and Simultaneous Orthognathic Surgery Using Computer-Assisted Surgery. J. Maxillofac. Oral Surg..

[89] Jin X., Chi W. (2024). Clinical effect of digitalized designed and 3D-printed repositioning splints in the treatment of anterior displacement of temporomandibular joint disc. BMC Musculoskelet. Disord..

[90] Singh S., Mishra P., Yadav S., Dubey P., Sitlani M., Di Blasio M., Cicciù M., Minervini G. (2025). Accuracy Evaluation of Access Cavity Preparation between Guided Endodontics and Conventional Technique. Eur. J. Gen. Dent..

[91] Mercuri L.G., Neto M.Q., Pourzal R. (2022). Alloplastic temporomandibular joint replacement: Present status and future perspectives of the elements of embodiment. Int. J. Oral Maxillofac. Surg..

[92] Duret F. (1991). Dental CAD-CAM six years after the first presentation at the 1985 A.D.F. Congress. Actual. Odontostomatol..

[93] Oberoi G., Agis H. (2021). 3D Printing in Endodontics. Guided Endodontics.

[94] Abdellatif D., Çapar I., Sarah D., Iandolo A., Meyer C., Davide M. (2025). Access cavity in endodontics: Balancing precision, preservation, and clinical needs. J. Conserv. Dent. Endod..

[95] Ackerman S., Aguilera F.C., Buie J.M., Glickman G.N., Umorin M., Wang Q., Jalali P. (2019). Accuracy of 3-dimensional–printed Endodontic Surgical Guide: A Human Cadaver Study. J. Endod..

[96] Zhao D., Xie W., Li T., Wang A., Wu L., Kang W., Wang L., Guo S., Tang X., Xie S. (2023). New-designed 3D printed surgical guide promotes the accuracy of endodontic microsurgery: A study of 14 upper anterior teeth. Sci. Rep..

[97] Chauhan J., Ataide I., Fernandes M. (2021). Three-dimensional printing in endodontics: A review of literature. IP Indian J. Conserv. Endod..

[98] Strbac G.D., Schnappauf A., Giannis K., Bertl M.H., Moritz A., Ulm C. (2016). Guided Autotransplantation of Teeth: A Novel Method Using Virtually Planned 3-dimensional Templates. J. Endod..

[99] Verweij J.P., Jongkees F.A., Anssari Moin D., Wismeijer D., van Merkesteyn J.P.R. (2017). Autotransplantation of teeth using computer-aided rapid prototyping of a three-dimensional replica of the donor tooth: A systematic literature review. Int. J. Oral Maxillofac. Surg..

[100] Singh A.K., Khanal N., Acharya N., Hasan M.R., Saito T. (2022). What Are the Complications, Success and Survival Rates for Autotransplanted Teeth? An Overview of Systematic Reviews and Metanalyses. Healthcare.

[101] Sato M., Garcia-Sanchez A., Sanchez S., Chen I.P. (2021). Use of 3-dimensional–Printed Guide in Hemisection and Autotransplantation of a Fusion Tooth: A Case Report. J. Endod..

[102] Cabezon C., Aubeux D., Pérez F., Gaudin A. (2023). 3D-Printed Metal Surgical Guide for Endodontic Microsurgery (a Proof of Concept). Appl. Sci..

[103] Garcia-Sanchez A., Mainkar A., Ordonez Fernandez E., Sanchez Velasco S., Weinstein G. (2019). 3D-printed guide for endodontic surgery. Clin. Dent. Rev..

[104] Giacomino C.M., Ray J.J., Wealleans J.A. (2018). Targeted Endodontic Microsurgery: A Novel Approach to Anatomically Challenging Scenarios Using 3-dimensional-printed Guides and Trephine Burs-A Report of 3 Cases. J. Endod..

[105] Hawkins T.K., Wealleans J.A., Pratt A.M., Ray J.J. (2020). Targeted endodontic microsurgery and endodontic microsurgery: A surgical simulation comparison. Int. Endod. J..

[106] Farajollahi M., Dianat O., Gholami S., Saber Tahan S. (2023). Application of an Endodontic Static Guide in Fiber Post Removal from a Compromised Tooth. Case Rep. Dent..

[107] Anderson J., Wealleans J., Ray J. (2018). Endodontic applications of 3D printing. Int. Endod. J..

[108] Wu Y., Huang L., Ge B., Zhang Y., Zhang J., Xie H., Zhu Y., Chen C. (2025). Application of 3D-printed resin guides for the removal of molar fiber posts. J. Dent..

[109] Choudhury S.A., Pal S., Sen S., Srivastava S.K., Sikdar C. (2025). 3D Printing and Guided Endodontics: The Future of Precision Access Cavity Design. Cureus.

[110] Connert T., Zehnder M., Amato M., Weiger R., Kühl S., Krastl G. (2018). Microguided Endodontics: A method to achieve minimally invasive access cavity preparation and root canal location in mandibular incisors using a novel computer-guided technique. Int. Endod. J..

[111] Urbone E.M., Tusas P., Gendviliene I., Rutkunas V., Drukteinis S. (2025). Accuracy of Dynamic Navigation vs. Freehand Endodontic Access Cavity Preparation in 3-Dimensionally Printed Teeth with Severe Pulp Canal Calcification. J. Funct. Biomater..

[112] Vijay D.S., Jayakumar P., Chatterjee D.D., Kapoor D.A., Dad D.J. (2025). 3D Printing in Periodontics—A Review. Univ. J. Dent. Sci..

[113] He L., Liu L., Li T., Zhuang D., Dai J., Wang B., Bi L. (2021). Exploring the imbalance of periodontitis immune system from the cellular to molecular level. Front. Genet..

[114] Sufaru I.G., Macovei G., Stoleriu S., Martu M.A., Luchian I., Kappenberg-Nitescu D.C., Solomon S.M. (2022). 3D Printed and Bioprinted Membranes and Scaffolds for the Periodontal Tissue Regeneration: A Narrative Review. Membranes.

[115] Chen H., Wang Y., Lai Y., Meng C., Ning X., Xu T., Song G., Zhang Y., Lin Y., Han B. (2025). Advances of 3D bioprinting technology for periodontal tissue regeneration. iScience.

[116] Acharya J.R., Kumar S., Girdhar G.A., Patel S., Parekh N.H., Patadiya H.H., Zinjala A.N., Haque M. (2025). 3D Bioprinting: Shaping the Future of Periodontal Tissue Regeneration and Disease Management. Cureus.

[117] Davidopoulou S., Karakostas P., Batas L., Barmpalexis P., Assimopoulou A., Angelopoulos C., Tsalikis L. (2024). Multidimensional 3D-Printed Scaffolds and Regeneration of Intrabony Periodontal Defects: A Systematic Review. J. Funct. Biomater..

[118] Mahardawi B., Jiaranuchart S., Arunjaroensuk S., Dhanesuan K., Mattheos N., Pimkhaokham A. (2025). The clinical benefit of alveolar ridge preservation in the posterior maxilla: A systematic review and meta-analysis. Sci. Rep..

[119] Davidopoulou S., Batas L., Karakostas P., Tortopidis D., Barmpalexis P., Assimopoulou A., Angelopoulos C., Tsalikis L. (2024). Multidimensional 3D-Printed Scaffolds for Ridge Preservation and Dental Implant Placement: A Systematic Review. Appl. Sci..

[120] Lee H., Kim E.Y., Lee U.L. (2023). Vertical augmentation of a severely atrophied posterior mandibular alveolar ridge for a dental implant using a patient-specific 3D printed PCL/BGS7 scaffold: A technical note. J. Stomatol. Oral Maxillofac. Surg..

[121] Mangano C., Giuliani A., De Tullio I., Raspanti M., Piattelli A., Iezzi G. (2021). Case report: Histological and histomorphometrical results of a 3-D printed biphasic calcium phosphate ceramic 7 years after insertion in a human maxillary alveolar ridge. Front. Bioeng. Biotechnol..

[122] Goh B.T., Teh L.Y., Tan D.B., Zhang Z., Teoh S.H. (2015). Novel 3D polycaprolactone scaffold for ridge preservation--a pilot randomised controlled clinical trial. Clin. Oral Implant. Res..

[123] Lim H.C., Kim S., Kim D.H., Herr Y., Chung J.H., Shin S.I. (2021). Factors affecting maxillary sinus pneumatization following posterior maxillary tooth extraction. J. Periodontal Implant. Sci..

[124] Yen H.H., Stathopoulou P.G. (2018). CAD/CAM and 3D-Printing Applications for Alveolar Ridge Augmentation. Curr. Oral Health Rep..

[125] Mangano C., Barboni B., Valbonetti L., Berardinelli P., Martelli A., Muttini A., Bedini R., Tetè S., Piattelli A., Mattioli M. (2015). In Vivo Behavior of a Custom-Made 3D Synthetic Bone Substitute in Sinus Augmentation Procedures in Sheep. J. Oral Implantol..

[126] Somji S.H., Valladares A., Ho Kim S., Cheng Paul Yu Y., Froum S.J. (2017). The use of 3D models to improve sinus augmentation outcomes—A case report. Singap. Dent. J..

[127] Mangano F.G., De Franco M., Caprioglio A., Macchi A., Piattelli A., Mangano C. (2014). Immediate, non-submerged, root-analogue direct laser metal sintering (DLMS) implants: A 1-year prospective study on 15 patients. Lasers Med. Sci..

[128] Gattani D., Kar N., Sahu J. (2020). 3D Printing-Periodontal Perspective. Int. J. Sci. Res..

[129] Anssari Moin D., Hassan B., Wismeijer D. (2016). A novel approach for custom three-dimensional printing of a zirconia root analogue implant by digital light processing. Clin. Oral Implant. Res..

[130] Konda P., Rafiq A.M. (2024). 3D printing: Changing the landscape of orthodontics. IP Indian J. Orthod. Dentofac. Res..

[131] Sassani F., Elmajian A., Roberts S. (1995). Computer-assisted fabrication of orthodontic appliances: Considering the possibilities. J. Am. Dent. Assoc..

[132] Rajagopalan A., Verma S., Kumar V., Verma R.K., Singh S.P. (2024). Accuracy of 3D Printing in Orthodontics: A Systematic Review and Meta-analysis. J. Indian Orthod. Soc..

[133] Dang W., Zheng H., Song G., Liang W., Han B. (2025). A Comparative Analysis of Plaster Model Preparation and Intraoral 3D Scanning Techniques in Orthodontic Dental Arch Modeling. Niger. J. Clin. Pract..

[134] Brown G.B., Currier G.F., Kadioglu O., Kierl J.P. (2018). Accuracy of 3-dimensional printed dental models reconstructed from digital intraoral impressions. Am. J. Orthod. Dentofac. Orthop..

[135] Rajasekaran A., Chaudhari P. (2023). Integrated manufacturing of direct 3D-printed clear aligners. Front. Dent. Med..

[136] Sycińska-Dziarnowska M., Szyszka-Sommerfeld L., Woźniak K., Lindauer S.J., Spagnuolo G. (2022). Predicting Interest in Orthodontic Aligners: A Google Trends Data Analysis. Int. J. Environ. Res. Public Health.

[137] Bae B.G., Kim Y.H., Lee G.H., Lee J., Min J., Kim H., Shin J.W., Chae H.S. (2025). A study on the compressive strength of three-dimensional direct printing aligner material for specific designing of clear aligners. Sci. Rep..

[138] Niu C., Li D., Zhang Y., Wang Y., Ning S., Zhao G., Ye Z., Kong Y., Yang D. (2024). Prospects for 3D-printing of clear aligners—A narrative review. Front. Mater..

[139] Stoev Y.Y., Uzunov T.T., Stoyanova N.S., Grozdanova-Uzunova R.G., Kosturkov D.N., Taneva I.K. (2023). Mechanical properties of materials for 3D printed orthodontic retainers. Folia Med..

[140] Firlej M., Zaborowicz K., Zaborowicz M., Firlej E., Domagała I., Pieniak D., Igielska-Kalwat J., Dmowski A., Biedziak B. (2022). Mechanical Properties of 3D Printed Orthodontic Retainers. Int. J. Environ. Res. Public Health.

[141] Beretta M., Mangano A., Gianolio A., Negrini S., Canova F.F., Cirulli N. (2021). A Fully Digital Workflow for PEEK Fixed Retainers. J. Clin. Orthod..

[142] Cole D., Bencharit S., Carrico C.K., Arias A., Tüfekçi E. (2019). Evaluation of fit for 3D-printed retainers compared with thermoform retainers. Am. J. Orthod. Dentofac. Orthop..

[143] Win P.P., Lai S.-Y., De-Shing Chen D., Sainbayar B., Peng T.-Y., Hsin-Chung Cheng J. (2025). Evaluation of the physical and mechanical properties of 3D-printed resin for orthodontic fixed lingual retainers: An in vitro study. J. Dent. Sci..

[144] Panayi N.C. (2023). In-office Customized Brackets: Aligning with the Future. Turk. J. Orthod..

[145] Panayi N. (2023). 3D printing of in office custom-made brackets: Concept, Design, Production and Evidence. Semin. Orthod..

[146] Nguyen V.A., Ha T.M.A. (2025). Esthetic orthodontic management with 3D-printed customized brackets for a Class II malocclusion with severe arch-length discrepancy: A case report. Medicine.

[147] Alam M.K., Hajeer M.Y., Alshammari A.H., Alenezi Z.M.M., Aldhafeeri S.B. (2024). Evaluation of the Efficacy of 3D-Printed Customized Orthodontic Brackets in Reducing Treatment Time and Improving Outcomes. J. Pharm. Bioallied Sci..

[148] Hanson M.S., Ontiveros J.C., English J.D., Wirthlin J.O., Cozad B.E., Harrington D.A., Kasper F.K. (2023). Effect of Material and Pad Abrasion on Shear Bond Strength of 3D-Printed Orthodontic Brackets. Orthod. Craniofac. Res..

[149] Sabbagh H., Hoffmann L., Wichelhaus A., Kessler A. (2025). Influence of the design of 3D-printed indirect bonding trays and experience of the clinician on the accuracy of bracket placement. J. Orofac. Orthop..

[150] Bachour P., Klabunde R., Gruenheid T. (2022). Transfer accuracy of 3D-printed trays for indirect bonding of orthodontic brackets. Angle Orthod..

[151] Singh D., Tripathi T., Rai P. (2024). 3D nasoalveolar moulding: A complete digital workflow model. Int. J. Sci. Res. Arch..

[152] Ahsanuddin S., Ahmed M., Slowikowski L., Heitzler J. (2022). Recent Advances in Nasoalveolar Molding Therapy Using 3D Technology. Craniomaxillofac. Trauma Reconstr..

[153] Puneet B., Gribel B.F., Aditya T., Garima A. (2025). Clinical Efficacy of Nasoalveolar Moulding Using Aligner NAM During Presurgical Infant Orthopaedics (PSIO) in Infants With Unilateral Cleft Lip and Palate (UCLP): A Retrospective Study. Orthod. Craniofac. Res..

[154] Yu Q., Gong X., Shen G. (2013). CAD presurgical nasoalveolar molding effects on the maxillary morphology in infants with UCLP. Oral Surg. Oral Med. Oral Pathol. Oral Radiol..

[155] Shaheen S.R., Sridevi E., Sai Sankar A., Krishna V., Sridhar M., Sankar K.S. (2023). Contemporary era of Three-dimensional printing in pediatric dentistry: An overview. J. Oral Res. Rev..

[156] Aktaş N., Ciftci V. (2024). Current applications of three-dimensional (3D) printing in pediatric dentistry: A literature review. J. Clin. Pediatr. Dent..

[157] Wairokpam B.D., Hage O., Angom A., Yumnam E. (2025). 3D printing in pediatric dentistry. J. Med. Soc..

[158] Aktaş N., Atabek D., Tunç O. (2025). Innovative 3D-printed educational models for vital pulp treatments and local anesthesia training in pediatric dentistry. BMC Med. Educ..

[159] Mello W.M.d., Dutra V., Liedke G.S. (2025). 3D Printing in Dental Education: A Scoping Review. Med. Princ. Pract..

[160] Tsai A.Y., Greene A.C. (2024). 3D printing in pediatric surgery. Semin. Pediatr. Surg..

[161] Les A.S., Ohye R.G., Filbrun A.G., Ghadimi Mahani M., Flanagan C.L., Daniels R.C., Kidwell K.M., Zopf D.A., Hollister S.J., Green G.E. (2019). 3D-printed, externally-implanted, bioresorbable airway splints for severe tracheobronchomalacia. Laryngoscope.

[162] Banerjee S., Jedika S., Sikha S., Samanta U., Meenakshi M. (2024). 3D printing: A comforting prospect in pediatric dentistry. Int. J. Adv. Res..

[163] Song J.-S., Shin Y., Kim J.-H., Lee K.E., Lee J., Min J., Kim H., Song J.S. (2025). Mechanical performance of 3D-printed and milled resins for pediatric provisional restorations. Sci. Rep..

[164] Agrawal P.V., Lahoti K.R., Rathi N., Jagtap A., Tasgaonkar A., Kotnis R. (2025). 3D Printed Denture for a Pediatric Patient with Complete Anodontia: A Case Report. Int. J. Clin. Pediatr. Dent..

[165] Afritha N., Sharanya, Moses J. (2022). 3-D Printed space maintainers—A review: Review Article. Int. J. Pedod. Rehabil..

[166] Watson L., Danley B., Versluis A., Tantbirojn D., Brooks J., Wells M.H. (2023). A Structural Analysis of 3D Printed Pediatric Space Maintainers. Pediatr. Dent..

[167] Thakur B., Bhardwaj A., Luke A.M., Wahjuningrum D.A. (2024). Effectiveness of traditional band and loop space maintainer vs 3D-printed space maintainer following the loss of primary teeth: A randomized clinical trial. Sci. Rep..

[168] Agarwal S., Mistry L.N., Kamath S., Thorat R., Gupta B., Kondkari S. (2025). Pioneering the Future of Oral Healthcare: Bioprinting and Its Transformative Clinical Potential in Dentistry. Cureus.

[169] Yücer S., Sarac B., Ciftci F. (2025). Bioprinting revolution: Innovative design of 3D bioactive scaffolds for living organs and transdermal tissues. Bioeng. Transl. Med..

[170] Olawade D.B., Leena N., Egbon E., Rai J., Mohammed A., Oladapo B.I., Boussios S. (2025). AI-Driven Advancements in Orthodontics for Precision and Patient Outcomes. Dent. J..

[171] Javaid M., Haleem A., Singh R.P., Rab S., Suman R., Kumar L. (2022). Significance of 4D printing for dentistry: Materials, process, and potentials. J. Oral Biol. Craniofac. Res..

[172] Alam M.K., Hajeer M.Y., Alserhani E.D.M., Albuhiran T.O., Alzhrani A.A., Alsalem A.M., Aljudaya S.A., Alruwaili H.H. (2025). Use of 4D Printing in Orthodontics: Self-Adjusting Brackets for Dynamic Tooth Movement. J. Pharm. Bioallied Sci..

[173] Moghanian A., Asadi P., Akbari M., Mohammad Aliha M.R., Kizilkurtlu A.A., Akpek A., Safaee S. (2025). New trends in 3D and 4D printed dental and orthopedic Implants: Methods, applications and future directions. Bioprinting.

[174] Huang T.K., Yang C.H., Hsieh Y.H., Wang J.C., Hung C.C. (2018). Augmented reality (AR) and virtual reality (VR) applied in dentistry. Kaohsiung J. Med. Sci..

[175] Basmacı F., Yonetken A.B., Bulut A.C., Cagiltay N.E. (2024). Educational potential of 3D teeth models in virtual reality. Int. Dent. J..

[176] Monalisa S., Alipuor M., Paul D., Rahman M.A., Siddika N., Apu E.H., Mostafiz R.B. (2025). Transforming Dental Care, Practice and Education with Additive Manufacturing and 3D Printing: Innovations in Materials, Technologies, and Future Pathways. Dent. J..

[177] Jun M.-K., Kim J.-W., Ku H.-M. (2025). Three-Dimensional Printing in Dentistry: A Scoping Review of Clinical Applications, Advantages, and Current Limitations. Oral.

[178] Packaeser M.G., de Oliveira Dal Piva A.M., Kleverlaan C.J., Mendes Tribst J.P. (2026). Economic aspects of 3D printing in restorative dentistry: A scoping review. Minerva Dent. Oral. Sci..

[179] Popescu M., Perieanu V.S., Burlibașa M., Vorovenci A., Malița M.A., Petri D.-C., Ștețiu A.A., Costea R.C., Costea R.M., Burlibașa A. (2025). Comparative Cost-Effectiveness of Resin 3D Printing Protocols in Dental Prosthodontics: A Systematic Review. Prosthesis.

